# Segmentation-Based Multi-Class Detection and Radiographic Charting of Periodontal and Restorative Conditions on Bitewing Radiographs Using Deep Learning

**DOI:** 10.3390/diagnostics16020322

**Published:** 2026-01-19

**Authors:** Ali Batuhan Bayırlı, Buse Kesgin, Mehmetcan Uytun, Alican Kuran, Mesude Çitir, Muhammet Burak Yavuz, Sevda Kurt Bayrakdar, Özer Çelik, İbrahim Şevki Bayrakdar, Kaan Orhan

**Affiliations:** 1 Department of Periodontology, Faculty of Dentistry, Mugla Sıtkı Kocman University, 48000 Mugla, Türkiye; mehmetcanuytun@mu.edu.tr; 2 Department of Restorative Dentistry, Faculty of Dentistry, Mugla Sıtkı Kocman University, 48000 Mugla, Türkiye; busekesgin@mu.edu.tr; 3 Department of Dentomaxillofacial Radiology, Faculty of Dentistry, Kocaeli University, 41380 Kocaeli, Türkiye; alican.kuran@kocaeli.edu.tr; 4 Department of Dentomaxillofacial Radiology, Faculty of Dentistry, Tokat Gaziosmanpaşa University, 60250 Tokat, Türkiye; mesude.citir@gop.edu.tr; 5 Department of Periodontology, Faculty of Dentistry, Eskişehir Osmangazi University, 26040 Eskişehir, Türkiye; muhammetburak.yavuz@ogu.edu.tr (M.B.Y.); sevda.kurtbayrakdar@ogu.edu.tr (S.K.B.); 6 Department of Mathematics and Computer Science, Faculty of Science, Eskişehir Osmangazi University, 26040 Eskişehir, Türkiye; ozer@ogu.edu.tr; 7 Department of Oral and Maxillofacial Radiology, Faculty of Dentistry, Eskişehir Osmangazi University, 26040 Eskişehir, Türkiye; isbayrakdar@ogu.edu.tr; 8 Department of Oral and Maxillofacial Radiology, Faculty of Dentistry, Ankara University, 06560 Ankara, Türkiye; knorhan@dentistry.ankara.edu.tr

**Keywords:** radiography, bitewing, deep learning, artificial intelligence, computer-aided diagnosis, alveolar bone loss, dental caries

## Abstract

**Background/Objective:** Bitewing radiographs are widely used for evaluating dental caries, restorations, and periodontal status due to their low radiation dose and high image quality. While artificial intelligence–based studies are common for other dental imaging modalities, multi-class diagnostic charting on bitewing radiographs remains limited. This study aimed to simultaneously detect eight periodontal and restorative parameters using a YOLOv8x-seg–based deep learning model and to assess its diagnostic performance. **Materials and Methods:** A total of 1197 digital bitewing radiographs were retrospectively analyzed and annotated by experts, resulting in 7860 labels across eight conditions. Periodontal conditions included alveolar bone loss, dental calculus, and furcation defects, while restorative and dental conditions comprised caries, cervical marginal gaps, open contacts, overhanging fillings, and secondary caries. The dataset was divided on a patient basis into training (80%), validation (10%), and test (10%) sets. The YOLOv8x-seg model was trained for 800 epochs with extensive data augmentation, and performance was evaluated using precision, recall, and F1-score, along with confusion matrices. **Results:** The model showed the highest accuracy in the alveolar bone loss class (precision: 0.84, recall: 0.93, F1: 0.88). While moderate performance was achieved for dental calculus (F1: 0.58) and caries (F1: 0.57) detection, lower scores were recorded in low-frequency classes such as cervical marginal gap (F1: 0.23), secondary caries (F1: 0.29), overhanging filling (F1: 0.35), furcation defect (F1: 0.40), and open contact (F1: 0.41). The overall segmentation performance achieved an mAP@0.5 of 0.30 and an mAP@0.5:0.95 of 0.10, indicating an acceptable performance level for segmentation-based multi-class models. **Conclusions:** The obtained findings demonstrate that the YOLOv8x-seg architecture can detect and segment periodontal conditions with high success and restorative parameters with moderate success in automation processes in bitewing radiographs. Accordingly, the model presents a methodologically feasible framework for the multiple and simultaneous radiographic evaluation of periodontal and restorative findings on bitewing radiographs, with performance varying across classes and lower sensitivity observed in low-frequency conditions.

## 1. Introduction

Radiographic imaging is a fundamental tool in dentistry for diagnosis, treatment planning, and the determination of disease prognosis. Conditions such as dental caries, faulty restorations, and alveolar bone loss, which cannot be fully identified through intraoral examination alone, often rely on radiographic assessment for accurate detection [1,2,3]. Bitewing radiographs have high diagnostic value in the evaluation of proximal caries, alveolar bone levels, and the marginal adaptation of restorations [4]. Owing to their low radiation dose and high spatial resolution, they are considered a standard diagnostic method for periodontal and restorative assessments. However, the interpretation of these radiographs depends on the clinician’s experience, and interobserver variability, visual fatigue, and subjective evaluations may negatively affect diagnostic accuracy [5].

In recent years, artificial intelligence (AI) and deep learning–based image analysis approaches have led to significant advances in dental radiology [6]. In particular, convolutional neural networks (CNNs) have been used for the automated identification of dental anatomical structures and pathological changes due to their ability to learn complex visual features from large datasets [7]. The literature reports that CNNs achieve high accuracy in the detection of dental caries and alveolar bone loss, thereby providing time efficiency, objectivity, and consistency in the diagnostic process [8,9]. However, the vast majority of existing studies have focused on only a single radiographic parameter. In contrast, loss of proximal contact, restorative defects, the presence of dental calculus, furcation defects, and alveolar bone loss are interrelated conditions. The simultaneous evaluation of these conditions may allow for a more comprehensive detection of the early stages of periodontal disease and carious lesions. Studies that have assessed periodontal and restorative parameters concurrently on bitewing radiographs are limited in number, and most have been conducted with small sample sizes or single-center designs [10,11,12,13]. This indicates the presence of a significant knowledge gap regarding the capacity of AI-based systems to perform multidimensional assessments in periodontal and restorative analysis.

Advanced object detection and segmentation algorithms developed in recent years, particularly YOLO (You Only Look Once)–based CNN architectures, have demonstrated promising results in the automated analysis of multiple lesions on dental radiographs [6,10] Among these architectures, YOLOv8 exhibits superior performance compared with previous versions due to improved segmentation accuracy, higher processing speed, and optimized parameter efficiency [14]. The integration of AI systems into clinical dentistry has the potential to increase reproducibility by reducing observer dependency in diagnostic processes. In addition, by supporting clinician decision-making in routine clinical workflows, such systems may improve early diagnostic accuracy and help prevent erroneous or missed detections. Furthermore, they may provide time and resource efficiency in treatment planning [15,16,17]. In this context, the development of clinical decision support systems represents an important step, particularly for the early diagnosis of periodontal diseases and carious and restorative defects, as well as for monitoring treatment response. However, it should be acknowledged that certain periodontal and restorative findings evaluated on bitewing radiographs, such as furcation involvement, represent radiographic manifestations of clinically complex conditions [18,19]. Therefore, these targets should be considered within the inherent limitations of the imaging modality and interpreted as indicators based on predefined radiographic criteria rather than as definitive clinical diagnoses.

The aim of this study was to automatically detect proximal contact status, carious lesions, restorative marginal defects, dental calculus accumulations, furcation defects, and alveolar bone loss on bitewing radiographs using a CNN–based model, and to evaluate the performance of the developed model in identifying these parameters. The YOLOv8x-seg–based artificial intelligence model trained in this study has the potential to serve as a decision support component that can be integrated into clinical workflows and radiographic software following the completion of appropriate validation and regulatory evaluations.

## 2. Materials and Methods

### 2.1. Study Design and Data Collection Process

This study was conducted through the retrospective evaluation of bitewing radiographs obtained from the digital archive of the Faculty of Dentistry at Muğla Sıtkı Koçman University. All procedures were carried out in accordance with the principles of the Declaration of Helsinki. The research protocol was approved by the Non-Interventional Clinical Research Ethics Committee of İzmir Bakırçay University on 26 February 2025 (Decision No: 2078).

A total of 1197 digital bitewing radiographs were evaluated. All images were acquired using the same radiographic system with standardized exposure parameters of 60 kV, 7 mA, and an exposure time of 0.08 s. The radiographs were exported in DICOM format and anonymized by removing all personal identifiers prior to analysis. All images underwent a preliminary evaluation by two periodontology specialists, one restorative dentistry specialist, and dentomaxillofacial radiology specialist to ensure homogeneity of diagnostic image quality. Images deemed unsuitable were excluded by unanimous consensus. This process aimed to ensure both technical and radiographic consistency of the study dataset.

Radiographs meeting the following criteria were included in the study: (i) belonging to individuals aged 18 years or older; (ii) diagnostic image quality consistent with the European Guidelines on Radiation Protection in Dental Radiology [20]; and (iii) clear visualization of all teeth and interproximal areas without artifacts. Radiographs were excluded if they exhibited: (i) pronounced motion artifacts, positioning errors, or sensor misalignment; (ii) severe beam scattering or distortion caused by metallic restorations, orthodontic brackets, or other radiopaque materials; or (iii) images compromising standardization due to anatomical variations, pathological lesions, or syndromic findings.

Additionally, an inter-examiner agreement analysis was performed before finalizing the dataset. For this purpose, Cohen’s kappa statistic was calculated based on binary categorical evaluations (suitable/unsuitable) independently performed by four evaluators. The resulting kappa coefficient exceeded 0.85 and, according to the criteria of Landis and Koch, was classified as indicating “almost perfect agreement” [21]. This confirmed strong methodological consistency among the evaluators.

### 2.2. Ground Truth

The annotation of the radiographs was performed by four researchers who evaluated the suitability of the images. Prior to the annotation process, a calibration training was provided by two researchers experienced in artificial intelligence applications in dentistry. Within the scope of this training, the researchers who would perform the annotations were given detailed instructions supported by annotated examples for the eight conditions to be labeled. The eight periodontal and restorative conditions evaluated during the annotation process were defined as alveolar bone loss, caries, cervical marginal gap, dental calculus, furcation defect, open contact, overhanging filling, and secondary caries.

Alveolar bone loss was defined as the presence of an interdental alveolar bone level positioned apical to the expected level in a healthy periodontium, with imaginary reference lines passing through the cementoenamel junctions of adjacent teeth and the alveolar bone crest remaining parallel [22]. Caries was defined as the presence of radiolucent areas confined to the enamel or extending from the enamel into the dentin or pulp [23]. Cervical marginal gap was radiographically defined as a discrepancy between the tooth structure and a dental crown or restorative material in the cervical region, measured at an angle of 90 degrees to the crown margin [24]. Dental calculus was radiographically defined as irregularly contoured, radiopaque masses adherent to the tooth surface [25]. For furcation defects, the furcation areas of multirooted teeth were examined, and the presence of radiolucent areas compatible with bone resorption was considered indicative of a furcation defect [22]. Open contact was defined as the absence of proximal contact between two adjacent teeth or between a restored tooth and the adjacent tooth, with a radiographically visible interproximal space [26]. Overhanging filling was defined as the extension of restorative material beyond the boundaries of the cavity preparation [27]. Secondary caries was defined as the presence of a well-defined radiolucent area adjacent to the margins of an existing restoration, particularly in the proximal, occlusal, or cervical regions [28].

Following the calibration training, the annotations were independently performed by four researchers using the CranioCatch Labeling Module (CranioCatch, Eskişehir, Türkiye), a web-based annotation platform specifically developed with a domain-oriented motivation for artificial intelligence applications in dentistry. Owing to the platform’s advanced annotation tools, each finding was marked in detail using polygonal segmentation. During the annotation phase, periodic weekly reviews were conducted by two senior experts to identify systematic deviations from the predefined criteria. Corrections were applied through expert consensus and used as feedback to maintain annotation consistency over time. After all annotations were completed, all images were re-evaluated by these two researchers in order to finalize them. In cases where consensus could not be reached between these two researchers, an independent expert external to the study was consulted to achieve agreement. In total, 7860 annotations were classified under eight categories. Each category was labeled by considering the radiographic diagnostic criteria defined in the literature and current clinical guidelines. Accordingly, the ground truth dataset created was prepared with a high level of accuracy, both clinically and technically, for use in model training.

### 2.3. Data Split

In the present study, the unit of analysis was the bitewing radiograph rather than the individual patient. Since multiple dental or periodontal conditions could be annotated within a single bitewing image, data splitting was performed on a radiograph basis rather than an annotation-based approach. All bitewing radiographs were anonymized prior to being uploaded to a web-based annotation platform to ensure patient confidentiality. Consequently, each bitewing radiograph was treated as an independent sample during dataset partitioning. The dataset was randomly sampled and divided into three subsets: the training set consisted of 959 images with 6545 annotations, the validation set comprised 119 images with 675 annotations, and the test set included 119 images with 640 annotations. This structure corresponds approximately to an 80% training, 10% validation, and 10% test split and is consistent with standard data partitioning strategies recommended in the literature for training CNN-based models. The validation set was used for hyperparameter optimization and for preventing model overfitting, whereas the test set was reserved for the independent and unbiased evaluation of model performance. As a result of this partitioning, a total of 7860 annotations were evenly distributed across the three subsets. The distribution of data for the eight different conditions following dataset splitting is presented in detail in Table 1, including both the number of radiographs and corresponding labels.

### 2.4. Development of Deep Learning Algorithm

The radiographs were subjected to pre-processing prior to analysis in accordance with the input requirements of the YOLOv8 architecture. The images were rescaled to a fixed input size of 640 pixels while preserving their original aspect ratio. In order to enhance the generalization capability of the model and to reduce the risk of overfitting, a comprehensive data augmentation strategy was applied during the training process. Without compromising dental anatomical integrity, noise-based augmentations such as Gaussian blur, CLAHE, Gaussian noise, ISO noise, and multiplicative noise were applied. In addition, image intensity variations were introduced through random brightness and contrast adjustments, and further augmentation techniques such as sharpening, random snow simulation, and sepia tone transformation were employed. Following these preprocessing and data augmentation steps, training of the YOLOv8x-seg model, which is the segmentation-optimized version of the YOLOv8 architecture, was initiated. The YOLOv8 algorithm is a modern deep learning architecture capable of performing object detection, instance segmentation, and classification tasks with high accuracy and efficiency. The YOLOv8 family consists of five different scales: YOLOv8n (nano), YOLOv8s (small), YOLOv8m (medium), YOLOv8l (large), and YOLOv8x (extra-large). As the architectural scale increases, the representational capacity and diagnostic performance of the model improve; however, computational cost and hardware requirements also increase accordingly. Smaller-scale models operate faster but provide relatively limited performance in multi-class segmentation tasks. Since the primary objective of this study was to perform multi-class segmentation of multiple periodontal and dental conditions in bitewing radiographs, higher accuracy and generalization performance were prioritized over processing speed, and therefore the YOLOv8x architecture was selected.

Architecturally, while YOLOv8 preserves the Backbone–Neck–Head structure, it incorporates significant improvements compared with previous versions to enhance gradient flow and computational efficiency. In the backbone section, a modified CSPDarknet-based structure is employed, where C2f (Cross Stage Partial with 2 fusion) modules are preferred instead of the C3 modules used in YOLOv5, allowing richer information flow. Additionally, the kernel size in the initial convolution layer has been optimized to 3 × 3, and a Spatial Pyramid Pooling Fusion (SPPF) module is utilized to capture multi-scale contextual features. In the neck section, Feature Pyramid Network (FPN) and Path Aggregation Network (PANet) structures are integrated to effectively fuse features obtained at different levels. In the head section, an anchor-free and decoupled structure is adopted, in which classification and bounding box regression tasks are separated to improve training stability. For the segmentation task, a structure inspired by the YOLACT approach is employed, where prototype masks generated via ProtoNet are combined with instance-specific mask coefficients to produce precise instance segmentation outputs.

The model was developed using the Python (version 3.10) programming language and the PyTorch (version 2.0) deep learning library. The training process was conducted using the computing infrastructure of the Faculty of Dentistry Dental-AI Laboratory at Eskişehir Osmangazi University, including a Dell PowerEdge T640 Compute Server, Dell PowerEdge T640 GPU Compute Server, and Dell PowerEdge R540 Storage Server (Dell Inc., Round Rock, TX, USA). The hyperparameters used during training were configured in accordance with the default settings recommended in the literature for YOLO-based segmentation models. Accordingly, the training duration was set to 800 epochs, the batch size to 8, and the initial learning rate to 0.01. The optimization process was carried out using the Stochastic Gradient Descent (SGD) algorithm, which is employed as the default optimizer in the YOLOv8 architecture. Loss functions were computed using the default loss structure provided by the Ultralytics YOLOv8 framework. Within this structure, Complete IoU (CIoU) loss was used for bounding box localization accuracy, Binary Cross-Entropy (BCE) loss for classification, and a model-specific mask loss function for segmentation masks, which were jointly optimized. To prevent overfitting during model selection, the early stopping technique was applied. The patience value was set to 50 epochs, and training was terminated when the validation mAP@0.5 metric showed no improvement for 50 consecutive epochs. Although the maximum number of epochs was set to 800, training was terminated at epoch 120 due to early stopping. The best validation performance was achieved at epoch 71, and the model weights from this epoch were selected as the final model. This strategy ensured that the final model utilized parameters that provided the best generalization performance without exhibiting overfitting.

**Figure 1:**
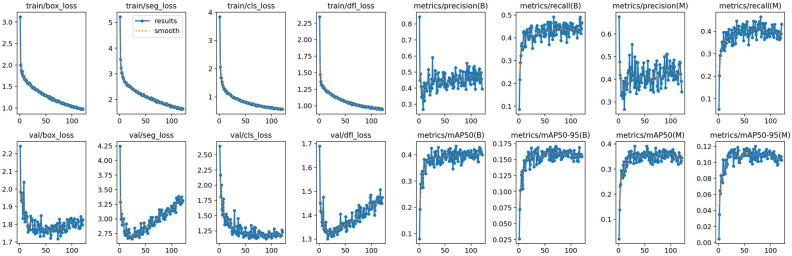
Training and validation loss and performance curves of the YOLOv8x-seg model across epochs.

The model’s training behavior, including loss and performance curves, is shown in Figure 1. The training loss decreased consistently throughout the learning process, indicating effective convergence and successful learning of the data. The validation loss reached a stable plateau after approximately the 30th epoch and remained stable thereafter, suggesting that the model preserved its generalization ability without signs of overfitting during the later stages of training.

### 2.5. Success Evaluation

The diagnostic accuracy and discriminative ability of the model were evaluated on an independent test set using multidimensional performance metrics. An object-based evaluation strategy was adopted. A predicted instance was considered a true positive only if its prediction of the model overlapped with the ground truth by at least 50% (IoU ≥ 0.50). Based on this criterion, the primary performance measures for object detection and segmentation tasks included precision, recall, F1 score, mAP@0.5 and mAP@0.5:0.95 were calculated. These metrics were calculated to quantitatively characterize the model’s performance at both the class-specific and overall accuracy levels.

The performance of the developed segmentation model was evaluated using the Dice Score and the Jaccard index, which quantify the degree of overlap between the predicted segmentation masks and the ground truth masks via pixel-based calculation. These metrics were calculated to objectively assess segmentation accuracy and boundary agreement. In addition to the segmentation model, the performance of the developed object detection model was evaluated using the Intersection over Union (IoU) metric, which measures the overlap between the predicted bounding boxes and the corresponding ground truth annotations.

In addition, both confusion matrices and normalized confusion matrices were generated to analyze classification performance and the distribution of errors. This evaluation enabled a more detailed analysis of the model’s behavior, particularly in categories with class imbalance. These confusion matrices were then used to calculate true positive rates (TPR) and false positive rates (FPR), from which ROC curves and corresponding AUC values were derived on an instance level for each class.

## 3. Results

**Figure 2:**
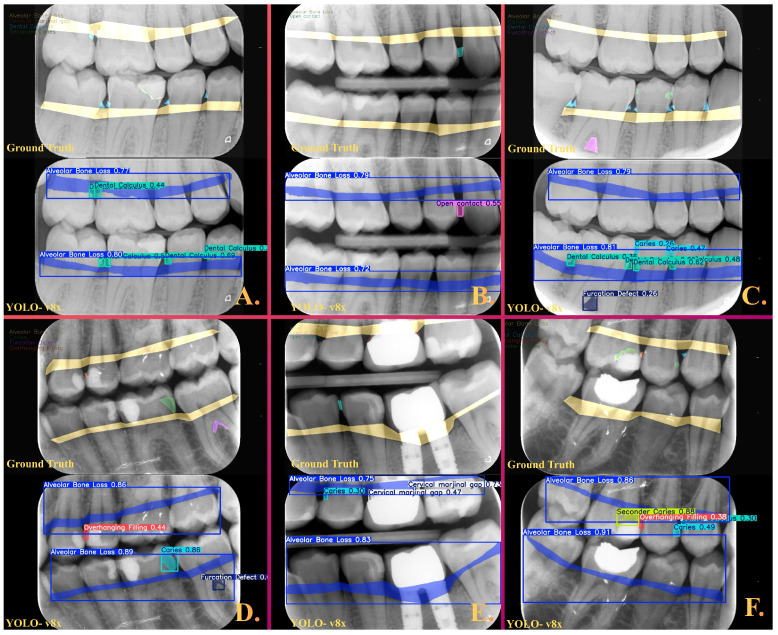
Representative images illustrating the performance of the YOLOv8x algorithm in segmenting and classifying eight different dental and periodontal conditions. (**A**) Total alveolar bone loss and dental calculus were successfully detected; however, secondary caries was not identified by the algorithm. (**B**) Total alveolar bone loss, together with an open contact condition, was segmented in agreement with the ground truth. (**C**) Total alveolar bone loss, calculus, caries, and a furcation defect were successfully segmented in accordance with the ground truth. (**D**) Alveolar bone loss, caries, a furcation defect, and an overhanging filling were accurately segmented in agreement with the ground truth. (**E**) The cervical marginal gap associated with tooth 26 was successfully segmented; however, the cervical marginal gap associated with tooth 25 was incorrectly classified as caries by the algorithm. (**F**) Secondary caries and an overhanging filling associated with tooth 16 were successfully segmented in agreement with the ground truth.

In this study, a total of 1197 bitewing radiographs were analyzed, and 7860 lesion labels were generated based on expert annotations. Examination of the class distribution revealed that the highest frequencies were observed in the caries (*n* = 2201) and alveolar bone loss (*n* = 2089) classes, whereas the lowest frequencies were found in the furcation defect (*n* = 128) and cervical marginal gap (*n* = 409) categories. The model demonstrated its highest performance in the alveolar bone loss class, with a precision of 0.84, recall of 0.93, and an F1-score of 0.88. The F1-score was 0.58 for the dental calculus class and 0.57 for the caries class, indicating a moderate level of performance in both categories. The lowest performance was observed in categories with the most pronounced class imbalance; the F1-score was 0.23 for cervical marginal gap, 0.29 for secondary caries, 0.35 for overhanging filling, 0.40 for furcation defect, and 0.41 for open contact. Representative images illustrating how the model segmented these dental and periodontal conditions and their comparison with the ground truth are presented in detail in Figure 2. In addition, performance metrics demonstrating the success of the multiclass model in segmenting all eight conditions are provided in detail in Table 1**.** Performance estimates for low-frequency classes should be interpreted with caution, as the limited number of test labels makes them highly sensitive to single-instance misclassifications. In addition to class-wise metrics, the overall segmentation performance of the model achieved an mAP@0.5 of 0.30 and an mAP@0.5:0.95 of 0.10 on the independent test set. Confusion matrix analysis showed that false-negative predictions constituted the dominant source of error across several low-frequency classes, with a substantial proportion of annotated lesions being classified as background.

The segmentation performance of the proposed model was evaluated for each class using the Dice Score and the Jaccard index. Among all classes, alveolar bone loss demonstrated the highest segmentation performance, achieving a Dice score of 0.64 and a Jaccard index of 0.54. Moderate segmentation performance was observed for caries, which yielded a Dice score of 0.44 and a Jaccard index of 0.34, followed by dental calculus with values of 0.44 and 0.32, respectively. Similarly, furcation defects and overhanging fillings recorded Dice scores of 0.42 and 0.40, along with Jaccard indices of 0.34 and 0.28. In contrast, lower segmentation performance was noted for cervical marginal gaps, open contacts and secondary caries. Cervical marginal gaps presented a Dice score of 0.25 and a Jaccard index of 0.17. Open contacts and secondary caries showed comparable results, reaching Dice values of 0.29 and 0.20, with corresponding Jaccard indices of 0.21 and 0.14. When all classes were considered together, the model achieved an aggregate Dice score of 0.38 and an overall Jaccard index of 0.29.

The detection performance was evaluated using the IoU metric. Alveolar bone loss demonstrated the highest spatial overlap, yielding an IoU score of 0.54. Moderate detection performance was observed for furcation defects and caries, which achieved IoU values of 0.34 and 0.34, respectively, followed by dental calculus at 0.32 and overhanging fillings at 0.28. Conversely, visually subtle findings resulted in lower localization scores; specifically, open contacts reached an IoU of 0.21, while cervical marginal gaps and secondary caries obtained scores of 0.17 and 0.14. Overall, the model achieved a mean IoU of 0.29 across all categories.

**Figure 3:**
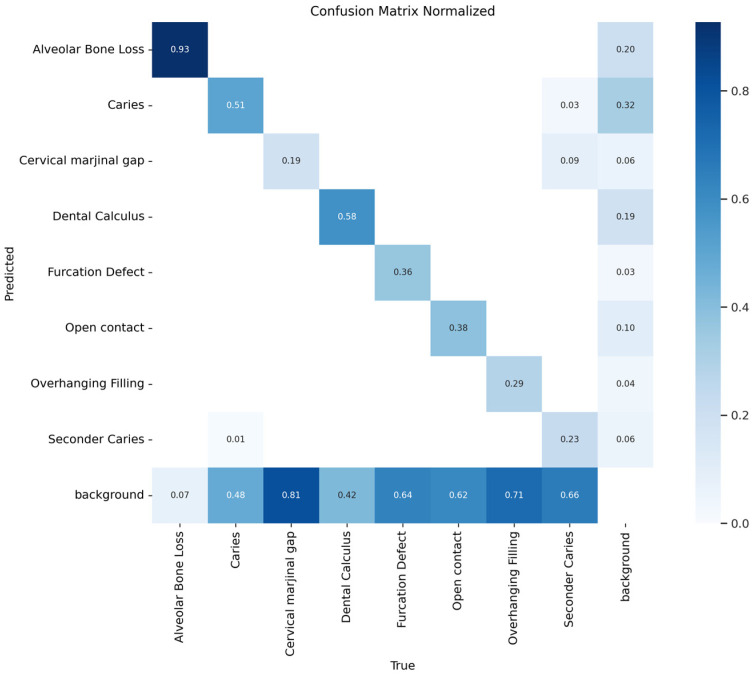
Confusion matrix illustrating the multiclass classification performance of the YOLOv8x model across eight dental and periodontal conditions.

**Figure 4:**
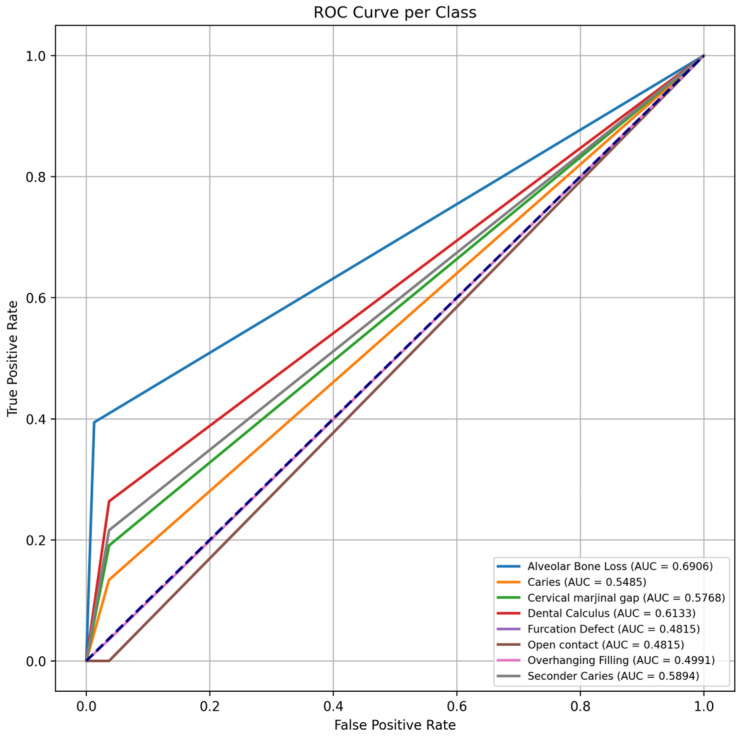
Area under the curve (AUC) values for the multiclass YOLOv8x model across eight dental and periodontal conditions, illustrating class-wise discriminative performance.

Confusion matrix analysis revealed that false negatives were predominant, particularly in classes with a limited number of samples, and that these categories were frequently confused with anatomically similar adjacent lesions. In contrast, the misclassification rate for the alveolar bone loss class was notably low, highlighting the model’s high discriminative capacity for this category. It was observed that the multiclass model experienced difficulty in directly detecting many dental and periodontal conditions and frequently failed to distinguish these lesions from the background class. The generally high rate of false-negative results is consistent with the findings presented in Table 1 and is also visually supported in Figure 3, where the background class is marked at high proportions. When the multiclass classification performance of the model was evaluated using AUC values, the highest performance was again observed for alveolar bone loss, with an AUC value of 0.6906. This was followed by dental calculus (AUC = 0.61) and secondary caries (AUC = 0.59), which demonstrated moderate discriminative ability. The caries and cervical marginal gap classes exhibited lower AUC values in the range of 0.55–0.58. For the furcation defect, open contact, and overhanging filling classes, AUC values remained below or near the 0.5 threshold, indicating limited discriminative performance for these complex pathologies (Figure 4).

## 4. Discussion

This study addresses a significant gap in artificial intelligence applications aimed at the simultaneous segmentation of periodontal and restorative parameters on bitewing radiographs by presenting one of the first comprehensive YOLOv8x-seg–based analyses to evaluate eight distinct clinical categories within a single model. While existing studies in the literature predominantly focus on a single radiographic parameter, the large-scale dataset used in this research and the ground truth approach based on a high level of expert consensus provide a methodological contribution toward the multidimensional evaluation of dental radiographs. In particular, the high performance observed for alveolar bone loss reflects the model’s strong segmentation capability for anatomically well-defined radiographic structures and should be interpreted as a descriptive performance outcome. Conversely, performance differences observed in categories characterized by pronounced class imbalance and morphological ambiguity suggest that advanced artificial intelligence approaches still have aspects that require further improvement to overcome radiographic challenges. Beyond algorithmic limitations, the achievable performance for certain categories may be constrained by label validity and radiographic observability. Some findings, such as furcation involvement, cervical marginal discrepancies, or subtle secondary caries, are inherently challenging to define consistently on bitewing radiographs, even among experienced clinicians. Consequently, model performance for these categories may be capped by the intrinsic ambiguity of the imaging modality rather than by architectural limitations alone.

The YOLOv8x-seg architecture employed in this study added substantial value to the research scope through its capability for simultaneous analysis in multi-lesion detection. The deep feature representation capacity of the CSP-based backbone and the multi-scale feature aggregation capability of the PAN-FPN structure contributed to high accuracy, particularly in radiographic parameters with large spatial extent and distinct morphology. However, the segmentation-centered design of the architecture resulted in more limited performance for parameters such as cervical marginal gap and secondary caries, which are small, low-contrast, or have indistinct boundaries. Nevertheless, the YOLOv8x-seg architecture, with its real-time processing capability and suitability for multi-lesion detection, provides a strong framework for the future integration of the model into clinical decision support systems. While architectural aspects of the YOLOv8x-seg framework are discussed to contextualize the technical feasibility of segmentation-based multiclass radiographic charting, several clinically oriented aspects, such as external validation, calibration of confidence scores, per-tooth or per-surface reporting, and the definition of clinically meaningful decision thresholds, were beyond the scope of the present study. These elements represent important directions for future research focused on clinical translation.

The multiclass annotation approach used in this study, together with the segmentation-focused structure of the YOLOv8x-seg architecture, provides a significant contribution to the clinically holistic evaluation of bitewing radiographs. The simultaneous detection of alveolar bone loss, carious lesions, defective restorations, dental calculus accumulations, and proximal contact loss, parameters that are traditionally assessed separately and often subjectively during conventional clinical examination, by a single artificial intelligence model demonstrates that periodontal and restorative radiographic examination processes can be optimized from an integrated perspective. In particular, the high level of accuracy achieved by the model in clinically important categories such as alveolar bone loss, caries, and dental calculus indicates a strong potential to increase diagnostic reproducibility in routine clinical workflows, reduce observer-dependent variability, and decrease the risk of missing early-stage lesions due to subjective assessment [29]. However, the performance limitations observed in categories with small sample sizes and morphologically indistinct boundaries indicate that such radiographic findings exhibit a high degree of observer dependency even in real clinical settings, and that the sensitivity of artificial intelligence models to these challenges is consistent with the existing literature [30]. Indeed, dental radiography studies employing multiclass YOLOv8 models have reported that, as the number of classes and lesion diversity increase, accuracy and F1 scores tend to decrease in certain categories, with distinct misclassification patterns particularly evident between morphologically similar or co-occurring classes [31,32]. These findings demonstrate that the methodological framework of the present study provides a strong foundation both for enhancing clinical diagnostic accuracy and for developing multidimensional decision support systems for dental radiography.

**Figure 5:**
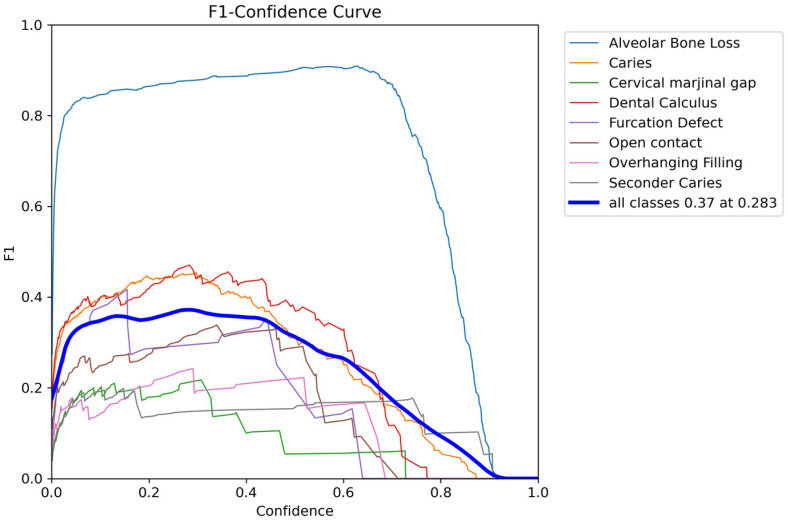
F1-Confidence curve illustrating the segmentation performance across eight different periodontal and dental conditions. The curves display the trade-off between the F1 score and the confidence threshold for each class. The bold blue line represents the aggregate performance for all classes, peaking at an F1 score of 0.37 at a confidence threshold of 0.283. Notably, ‘Alveolar Bone Loss’ demonstrates superior segmentation performance compared to other classes.

When the class-based diagnostic performance of the model is examined in detail, it is observed that the YOLOv8x-seg architecture achieves higher levels of accuracy in morphologically distinct classes with high radiographic contrast, whereas factors such as class imbalance, low contrast, and anatomical heterogeneity limit performance in certain categories. The high F1 score obtained for the alveolar bone loss class provided the model with a discernible structural advantage due to the lesion’s well-defined cortical boundaries and large segmentation area. The moderate performance observed for caries and dental calculus, which are smaller lesions with greater morphological variability, indicates that anatomical variation and intra-class heterogeneity complicate model training for these categories. In contrast, the low F1 scores observed in categories with limited sample sizes and less radiographically distinct boundaries, such as cervical marginal gap, secondary caries, furcation defect, and overhanging filling, demonstrate that class imbalance and annotation complexity have a pronounced impact on the model’s class-based sensitivity. Şener and Karacan (2025) reported that dataset size, class distribution, and annotation quality have a critical influence on model performance [33]. In our study, confusion matrix results showed that false negatives were predominant, particularly in low-frequency classes, and that these findings were most often confused with adjacent classes. The predominance of false-negative predictions and background-dominant outputs observed in the Results reflects the conservative behavior of the multiclass segmentation model under pronounced class imbalance and radiographic ambiguity. Consequently, the present framework should be interpreted as a methodological proof-of-concept for simultaneous radiographic charting rather than as a standalone screening or diagnostic system. At the same time, the clustering of optimal threshold values at moderate confidence scores in the F1–confidence analyses suggest that the model offers a clinician-adjustable decision mechanism, enhancing its usability in practical clinical scenarios (Figure 5). It has been reported in the literature that models containing a limited number of classes achieve higher F1 scores [34]. This indicates that a reduced number of classes and balanced data can artificially inflate performance metrics. Similarly, the high accuracy reported in the YOLOv8 study by Bayati et al., which targeted a single parameter, interproximal caries, supports the notion that classes with uniform morphology are more easily learned by the model [35]. In parallel with these studies, the YOLO-DentSeg model developed panoramic radiographs, which included only three classes, caries, deep caries, and impacted teeth, reported high segmentation accuracy within a problem structure characterized by relatively low morphological diversity. In the same study, it was noted that “jagged edges” and irregular contour generation observed in the segmentation masks produced by the compared models could limit segmentation quality and practical usability in complex anatomical structures [36]. Consistent with these findings, as observed in the five-class YOLOv8-based dental X-ray analysis by Aldanma et al., accuracy and F1 scores remain at moderate levels in scenarios with increased class numbers and morphological heterogeneity, highlighting the constraining effects of class imbalance and structural similarity on performance in multiclass dental AI models [31]. Therefore, in the present study, the requirement to simultaneously segment eight heterogeneous radiographic findings, along with pronounced anatomical overlap and high intra-class variation, makes the relatively lower F1 scores an expected outcome.

The model’s ability to achieve acceptable accuracy levels in segmentation-based multiclass detection demonstrates that the YOLOv8 architecture offers strong capacity for discriminating complex dental structures on bitewing radiographs. However, the pronounced performance differences between classes indicate that model sensitivity remains limited, particularly for lesions with low sample sizes and morphologically adjacent characteristics. The limitation observed in our study is consistent with previous findings reporting decreased performance in underrepresented categories in deep learning models in the presence of class imbalance and insufficient data representation [1]. Confusion matrix analysis showing the predominance of false negatives in low-frequency classes further indicates that class imbalance adversely affects the learning process and aligns with similar error patterns reported in the literature for multiclass dental AI models. In contrast, the low misclassification rate observed for the alveolar bone loss class supports the notion that distinct cortical contours are learned more consistently by the model. Studies focusing on the automated detection of periodontal bone loss on panoramic radiographs have reported that CNN models trained on a limited number of image segments can achieve accuracy levels comparable to experienced clinicians; however, limited dataset size has been identified as a constraining factor affecting model performance and generalizability [37]. Nevertheless, both these studies and more recent deep learning approaches emphasize that training dataset size and annotation quality are among the primary determinants of model performance and generalizability [11,38]. The clustering of optimal threshold values at moderate confidence levels in the F1–confidence analyses further indicates that the model possesses a decision structure adaptable to clinical scenarios (Figure 4). This finding demonstrates that the model can effectively evaluate intermediate probability levels associated with uncertainty, rather than relying exclusively on high-confidence scores. Consequently, the model is considered capable of exhibiting a more balanced and application-oriented performance profile in the presence of the variable image quality and heterogeneous parameter distributions commonly encountered in dental radiographs. In this context, studies such as Chau et al. demonstrate the clinical deployment of AI-based diagnostic tools following external validation [39]. By contrast, the present study addresses an earlier but essential methodological step by focusing on reliable multiclass segmentation and automated radiographic charting, which form the technical foundation for future clinically integrated decision support systems.

This study is one of the limited numbers of multiclass artificial intelligence applications focusing on the segmentation-based simultaneous evaluation of eight different periodontal and restorative parameters on bitewing radiographs, thereby providing a significant methodological contribution to the literature. The large sample size, the annotation framework based on a high level of expert consensus, and the use of an advanced deep segmentation architecture represent key methodological strengths that enhance the clinical reliability of the findings. In particular, the high accuracy achieved in anatomically well-defined categories supports the model’s potential for integration into clinical workflows. Nevertheless, the study has certain limitations. Class imbalance in low-frequency and morphologically ambiguous categories reduced model sensitivity. For classes represented by very small numbers of test annotations, class-wise metrics are statistically unstable and should be regarded as descriptive indicators rather than robust estimates of real-world performance. Class-wise performance metrics were reported as point estimates without confidence intervals. Due to pronounced class imbalance and limited test samples for several low-frequency conditions, uncertainty estimation was not feasible. Therefore, the results should be interpreted as descriptive, particularly for rare findings. The retrospective and single-center nature of the dataset may introduce selection bias and limit the generalizability of the results. The absence of an independent external validation dataset should be considered when interpreting the results. However, the use of standardized imaging protocols within a single institution ensured consistent image quality and reliable annotation, supporting robust internal model evaluation.

In addition, anatomical overlap and intra-class variation inherent to bitewing images led to performance fluctuations in segmentation masks, particularly for small or low-contrast lesions. The lack of systematic benchmarking against alternative deep learning architectures represents an additional limitation of the present study. From an ethical perspective, the use of retrospective datasets may introduce algorithmic bias, particularly affecting underrepresented patient groups or uncommon anatomical patterns, which should be considered in future fairness-aware and multicenter dental AI studies. Future studies focusing on multicenter datasets, advanced data augmentation strategies aimed at reducing class imbalance, and improvements in mask quality in anatomically complex regions may further strengthen the clinical applicability of the model.

## 5. Conclusions

This study provides a methodologically meaningful contribution by introducing a multiclass, segmentation-based framework for the simultaneous detection of eight periodontal and restorative parameters on bitewing radiographs. The model demonstrated its strongest performance in morphologically well-defined findings, particularly alveolar bone loss, supporting the feasibility of automated assessment for this clinically important parameter. In contrast, reduced performance in several low-frequency and morphologically ambiguous categories highlights class-dependent feasibility and underscores the need for larger, multicenter datasets and further refinement before broader clinical deployment.

## Figures and Tables

**Table 1:** Performance metrics of the multiclass YOLOv8x model for the segmentation and classification of eight dental and periodontal conditions on the independent test set. Precision, recall, F1-score, and related evaluation metrics are reported for each class.

Class	Train Image/Label Count	Validation Image/Label Count	Test Image/Label Count	TP	FP	FN	Precision	Recall	F1-Score
**Alveolar Bone Loss**	959/1671	119/211	119/207	192	37	15	0.83	0.92	0.88
**Caries**	959/1775	119/210	119/216	110	59	106	0.65	0.50	0.57
**Cervical Marginal Gap**	959/340	119/37	119/32	6	14	26	0.30	0.18	0.23
**Dental Calculus**	959/658	119/71	119/79	46	34	33	0.57	0.58	0.57
**Furcation Defect**	959/101	119/16	119/11	4	5	7	0.44	0.36	0.40
**Open contact**	959/420	119/37	119/39	15	19	24	0.44	0.38	0.41
**Overhanging Filling**	959/208	119/39	119/21	6	7	15	0.46	0.28	0.35
**Seconder Caries**	959/372	119/54	119/35	8	12	27	0.40	0.22	0.29

## Data Availability

The data presented in this study are available on request from the corresponding author due to ethical and privacy.
